# Saturation of acyl chains converts cardiolipin from an antagonist to an activator of Toll-like receptor-4

**DOI:** 10.1007/s00018-019-03113-5

**Published:** 2019-05-06

**Authors:** Malvina Pizzuto, Caroline Lonez, Alberto Baroja-Mazo, Helios Martínez-Banaclocha, Panagiotis Tourlomousis, Monique Gangloff, Pablo Pelegrin, Jean-Marie Ruysschaert, Nicholas J. Gay, Clare E. Bryant

**Affiliations:** 1 Structure and Function of Biological Membranes, Université Libre de Bruxelles, Blvd du Triomphe Access 2, 1050 Brussels, Belgium; 2 Department of Biochemistry, University of Cambridge, 80 Tennis Court Road, Cambridge, CB2 1GA UK; 3 Department of Veterinary Medicine, University of Cambridge, Madingley Rd, Cambridge, CB3 0ES UK; 4 Molecular Inflammation Group, Biomedical Research Institute of Murcia IMIB-Arrixaca, Clinical University Hospital Virgen de la Arrixaca, Carretera Buenavista s/n, 30120 Murcia, Spain

**Keywords:** Cardiolipin, Toll-like receptor (TLR), TLR4-antagonist, TLR4-agonist, Inflammation resolution, Barth syndrome, Vaccine adjuvant, Anti-inflammatory

## Abstract

**Abstract:**

Cardiolipins (CLs) are tetra-acylated diphosphatidylglycerols found in bacteria, yeast, plants, and animals. In healthy mammals, CLs are unsaturated, whereas saturated CLs are found in blood cells from Barth syndrome patients and in some Gram-positive bacteria. Here, we show that unsaturated but not saturated CLs block LPS-induced NF-κB activation, TNF-α and IP-10 secretion in human and murine macrophages, as well as LPS-induced TNF-α and IL-1β release in human blood mononuclear cells. Using HEK293 cells transfected with Toll-like receptor 4 (TLR4) and its co-receptor Myeloid Differentiation 2 (MD2), we demonstrate that unsaturated CLs compete with LPS for binding TLR4/MD2 preventing its activation, whereas saturated CLs are TLR4/MD2 agonists. As a consequence, saturated CLs induce a pro-inflammatory response in macrophages characterized by TNF-α and IP-10 secretion, and activate the alternative NLRP3 inflammasome pathway in human blood-derived monocytes. Thus, we identify that double bonds discriminate between anti- and pro-inflammatory properties of tetra-acylated molecules, providing a rationale for the development of TLR4 activators and inhibitors for use as vaccine adjuvants or in the treatment of TLR4-related diseases.

**Graphical abstract:**

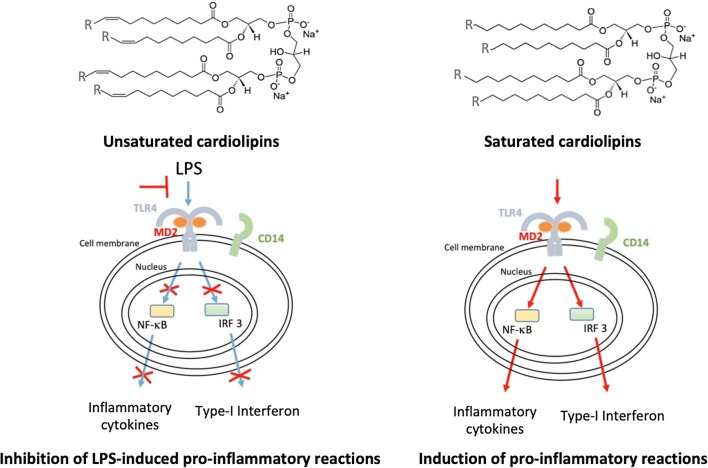

**Electronic supplementary material:**

The online version of this article (10.1007/s00018-019-03113-5) contains supplementary material, which is available to authorized users.

## Introduction

Cardiolipin (CL) is a tetra-acylated diphosphatidylglycerol found in yeast, bacteria, plants, and animals. Acyl chain length and degree of unsaturation vary depending on species, tissue, and pathological conditions [1–3]. Bacterial CL is highly heterogeneous, but some bacterial species like the Gram-positive *Micrococcus luteus* contain only saturated CL [2]. Thanks to the enzyme tafazzin that remodels CL after synthesis, saturated CLs are almost absent in mammals [2, 4]. However, in Barth syndrome (BTHS), genetic mutations of tafazzin induce a loss of selectivity leading to a decrease in unsaturated CL and an increase in CL with saturated chains, especially C16:0 [2]. Mammalian CL is located in the inner mitochondrial membrane where it permits cristae formation and increases the efficiency of respiratory supercomplexes and ATP-synthase [5–7]. Saturation of CL leads to defective mitochondrial bioenergetics and explains why BTHS patients have cardiac and skeletal myopathy. It is not known whether changes in CL saturation are also linked to the chronic inflammation and neutropenia in BTHS [8–10].

Unsaturated CL from bovine heart inhibits bacterial lipopolysaccharide (LPS)-induced pro-inflammatory cytokine production [11–13], which suggests a role for CL in regulating Toll-like receptors’ (TLRs) activation. TLRs are transmembrane proteins that recognize specific pathogen-associated molecular patterns. Soluble Lipid-Binding Protein (LBP) and the Cluster of Differentiation-14 (CD14) transfer LPS from bacteria to TLR4 and its co-receptor Myeloid Differentiation 2 (MD2). LPS binding to TLR4/MD2 heterodimer induces assembly of an active tetrameric complex that nucleates the formation of the Myddosome signaling hub. This, in turn, leads to Nuclear Factor-kappa B (NF-κB) and Interferon Regulatory Factor 3 (IRF3) activation to induce pro-inflammatory and type-I Interferon cytokine secretion [14–16]. The precise molecular mechanism by which TLR4 signaling is inhibited by CL remains to be demonstrated.

Unsaturated fatty acids are present in TLR4 antagonist such as the LPS synthesized by *Rhodobacter sphaeroides* (RS-LPS) or *Rhodobacter capsulatus* (RC-LPS) and the synthetic Eritoran [17–19]. This suggests that the unsaturation of the acyl chains is critical for TLR4 inhibition, although the heterogeneity of LPS extracted from bacteria and the lack of comparable synthetic LPS molecules has impeded a proper analysis. Despite the lack of a polysaccharide moiety, CLs may have a mechanism of action that is similar to LPS and act as agonists or antagonists depending on acyl chain composition. The ease of synthesis of homo-tetra-acylated diphosphatidylglycerols allowed us to design and obtain saturated CLs with 14, 16, and 18 chain length and their unsaturated counterparts. These CLs were used to investigate the mechanism of TLR4 inhibition and how saturation and acyl chain length influence the inflammatory activity of CLs that may underlie diseases such as BTHS.

## Materials and methods

### Reagents and cell lines

Human embryonic kidney cells were purchased from the American Type Culture Collection (293 [HEK293] (ATCC^®^ CRL1573™)) and human acute monocytic leukemia cell line (THP1 ECACC 88081201) was obtained from European Collection of Authenticated Cell Cultures. Immortalized bone marrow-derived macrophages (iBMDMs) from C57BL/6 wild-type (WT), immortalized by v-myc/v-raf recombinant retrovirus infection, were gifts from Dr Katherine Fitzgerald, University of Massachusetts Medical School, USA [20]. DMEM (Dulbecco’s Modified Eagle’s Medium) media, l-glutamine, sodium pyruvate, penicillin, and streptomycin were from Lonza. Phorbol 12-myristate 13-acetate (PMA) was from Sigma-Aldrich. Fetal bovine serum (FBS) from South America was from Lonza and Fetal Bovine Serum (FBS) from North America was purchased from Sigma-Aldrich. RAW-Blue™ cells, Zeocin™, ultrapure standard lipopolysaccharide (LPS) from *E. coli* 0111:B4, RS-LPS and Quanti-Blue™ were from InvivoGen. Tetrazolium dye MTT 3-(4,5-dimethylthiazol-2-yl)-2,5-diphenyltetrazolium bromide was from Sigma. Cardiolipins were purchased from Avanti Lipids.

### Primary bone marrow-derived macrophages (BMDMs)

Bone marrow was isolated from femurs and tibias of 10-week-old C57BL/6 mice killed by cervical dislocation. Briefly, bone marrow was flushed out with a syringe filled with DMEM, pelleted down, and resuspended and homogenized in complete medium (DMEM supplemented with 10% heat-inactivated FBS from South America, 2 mM glutamine, 50 U/mL penicillin, 50 μg/mL streptomycin, and 20% of supernatant taken from L929 cells). The cell suspension generated thereafter was then maintained in petri dishes for 7 days.

### Peripheral blood mononuclear cells’ (PBMCs) isolation

Blood from healthy volunteers was collected upon informed consent. PBMCs were obtained by Ficoll gradient centrifugation in Histopaque-1077 (Sigma-Aldrich, St Louis, Missouri, United States) using SepMate™ isolation tubes from STEMCELL™ according to the manufacturer’s instructions. Isolated PBMCs were resuspended in RPMI 1640 supplemented with 10% heat-inactivated FBS.

### Monocytes isolation

Human monocytes were isolated from PBMCs by immunomagnetic negative selection using the EasySep™ human monocyte enrichment kit without CD16 depletion (STEMCELL™ Technologies).

### Cell line cultures

HEK 293 cells were cultured in DMEM supplemented with 10% heat-inactivated FBS from North America, 2 mM l-glutamine, 1 mM sodium pyruvate, 50 U/mL penicillin, and 50 μg/mL streptomycin.

THP-1 cells were cultured in RPMI 1640 supplemented with 10% heat-inactivated FBS from South America, 2 mM l-glutamine, 1 mM sodium pyruvate, 50 U/mL penicillin, and 50 μg/mL streptomycin.

Immortalized BMDM cells were cultured in DMEM supplemented with 10% heat-inactivated FBS from South America, 2 mM l-glutamine, 1 mM sodium pyruvate, 50 U/mL penicillin, and 50 μg/mL streptomycin.

RAW-Blue™ cells are RAW 264.7 macrophages that stably express a secreted embryonic alkaline phosphatase (SEAP) gene inducible by NF-κB. They were cultured in DMEM supplemented with 10% heat-inactivated FBS from South America, 2 mM l-glutamine, 1 mM sodium pyruvate, 50 U/mL penicillin, 50 μg/mL streptomycin, and 200 μg/mL ZeocinTM.

All cells were incubated at 37 °C in a 5% CO_2_ atmosphere.

All cell lines were tested for mycoplasma contamination on a regular basis. To avoid divergence from the parent line, cell cultures were passaged up to ten times.

### Cardiolipin liposome preparation

All synthetic cardiolipins are homoacylated, and are referred to here as C14:0, C14:1, C16:0, C16:1, C18:0, and C18:1 CL. Heart CL is composed mainly of C18:2 homoacylated CL but contains up to 20% of the heteroacylated (C18:2)_3_(C18:1) cardiolipin. This means that at least 95% of acyl chains in heart CL correspond to C18:2 and we will, therefore, refer to this lipid as C18:2 CL in the present manuscript.

CLs were purchased as powder (C18:0 and C16:0) or as CHCl_3_ solution and stored at − 20 °C. CHCl_3_ solutions of CL 1 mg/mL were prepared and lipid films were formed by solvent evaporation under nitrogen stream, and vacuum dried overnight and kept at − 20 °C. Before each experiment, liposomes were freshly formed by resuspending lipid films into filtered Hepes 10 mM heated at 70 °C and sonicated for 5 min at medium power (BioRuptor, Diagenode).

### RAW-Blue™ cell experiments

RAW-Blue™ cells were seeded the day before the experiment at 5 × 10^5^ cells/mL in 48-well plates (500 μL/well).

These cells express a secreted embryonic alkaline phosphatase (SEAP) when NF-κB is activated. NF-κB activation was measured by measuring the alkaline phosphatase produced by RAW-Blue™ cells using the Quanti-Blue™ reagent (InvivoGen), according to the manufacturer’s instructions.

### HEK293 cell experiments

Cells were seeded at 4 × 10^4^ cells/mL in 96-well plates (200 μL/well) and transiently transfected 4 days later. Expression vectors containing an NF-κB transcription reporter vector-encoding firefly luciferase (10 ng/well pNF-κB-luc from Clontech), and a constitutively active reporter vector-encoding Renilla luciferase (5 ng/well phRG-TK; Promega), together with empty vector (pcDNA3.1 from Invitrogen Cat. N. V79020) and cDNA encoding human membrane CD14 (3 ng/well) and human or murine TLR4 and MD2 (3 ng/well) were mixed with jetPEI (Polyplus transfection Cat.N. 101-10N) and incubated with cells according to the manufacturer’s instructions. After 48 h, the medium was removed and cells treated as indicated in figures.

After treatment supernatants were discarded, cells were washed with PBS and lysed with passive lysis buffer (Promega Cat.N. E1941). Luciferase and Renilla activity on cell lysates were quantified on a BioTek Synergy HT microplate reader using home-made luciferase reagent (20 mM Tricine, 2.67 mM MgSO_4_·7H_2_O, 0.265 mM (MgCO_3_)4Mg(OH)_2_·5H_2_O, 0.1 mM EDTA, 33.3 mM DTT, 530 μM ATP, 270 μM Acetyl Coenzyme A (Lithium salt), 470 μM luciferin (Biosynth), pH 7.8, diluted two times in water before use), or coelenterazine (Biosynth) dissolved in ethanol at 1 mg/mL and diluted 500 times in PBS before use as previously described [21]. Firefly and Renilla luciferase activity on cell lysates were normalized, and data were expressed as fold induction as compared to unstimulated conditions.

### PBMCs, monocytes THP-1, and BMDM cell experiment

Two days prior to stimulation, THP-1 cells were seed at 3.5 × 10^6^ cells/mL (200 μL/well) and primed with 50 nM PMA to differentiate into macrophages [22].

Immortalized BMDM were seeded the day before the experiment at 5 × 10^5^ cells/mL in 48-well plates (500 μL/well).

Primary BMDM cells were detached from petri dishes and seeded the day before the experiment in 96-well plates at 0.75 × 10^6^ cells/mL (200 μL/well).

100.000 of freshly isolated PBMCs or monocytes were mixed with stimulants to a final volume of 160 μL/well in round bottom 96-well plates.

After stimulation, supernatants were collected, centrifuged, and assayed for cytokine secretion, while cells were assayed for viability.

### Viability (MTT) test

Cells were incubated 3 h with 0.5 mg/mL of MTT diluted in medium without phenol red. MTT, a yellow tetrazole, is reduced to purple formazan crystals in living cells. Plates were centrifuged, medium discarded, and crystals diluted in DMSO. Absorbance at 570 nm (corresponding to formazan crystal absorbance) and 620 nm (used as a reference) was read with a BioTek Synergy HT Microplate Readers and normalized with respect to cell control.

### Cytokine assays

TNF-α and IP-10 were quantified in cell supernatants using DuoSet ELISA kits from R&D Systems and human IL-1β using Instant ELISA™ Kit from Invitrogen, according to the manufacturer’s instructions. Absorbance was read with a BioTek Synergy HT Microplate Readers.

## Results

### Saturation of acyl chains deprives cardiolipins of their anti-inflammatory properties

**Fig. 1:**
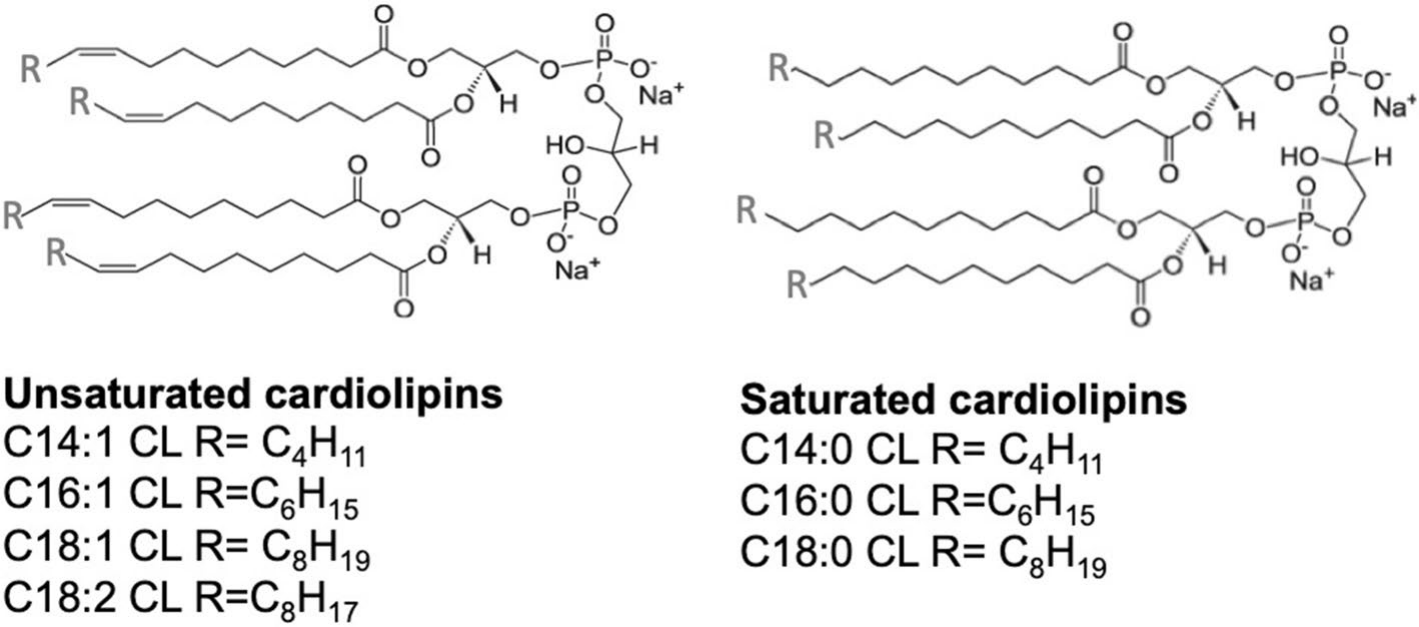
Structures of unsaturated and saturated cardiolipins

**Fig. 2:**
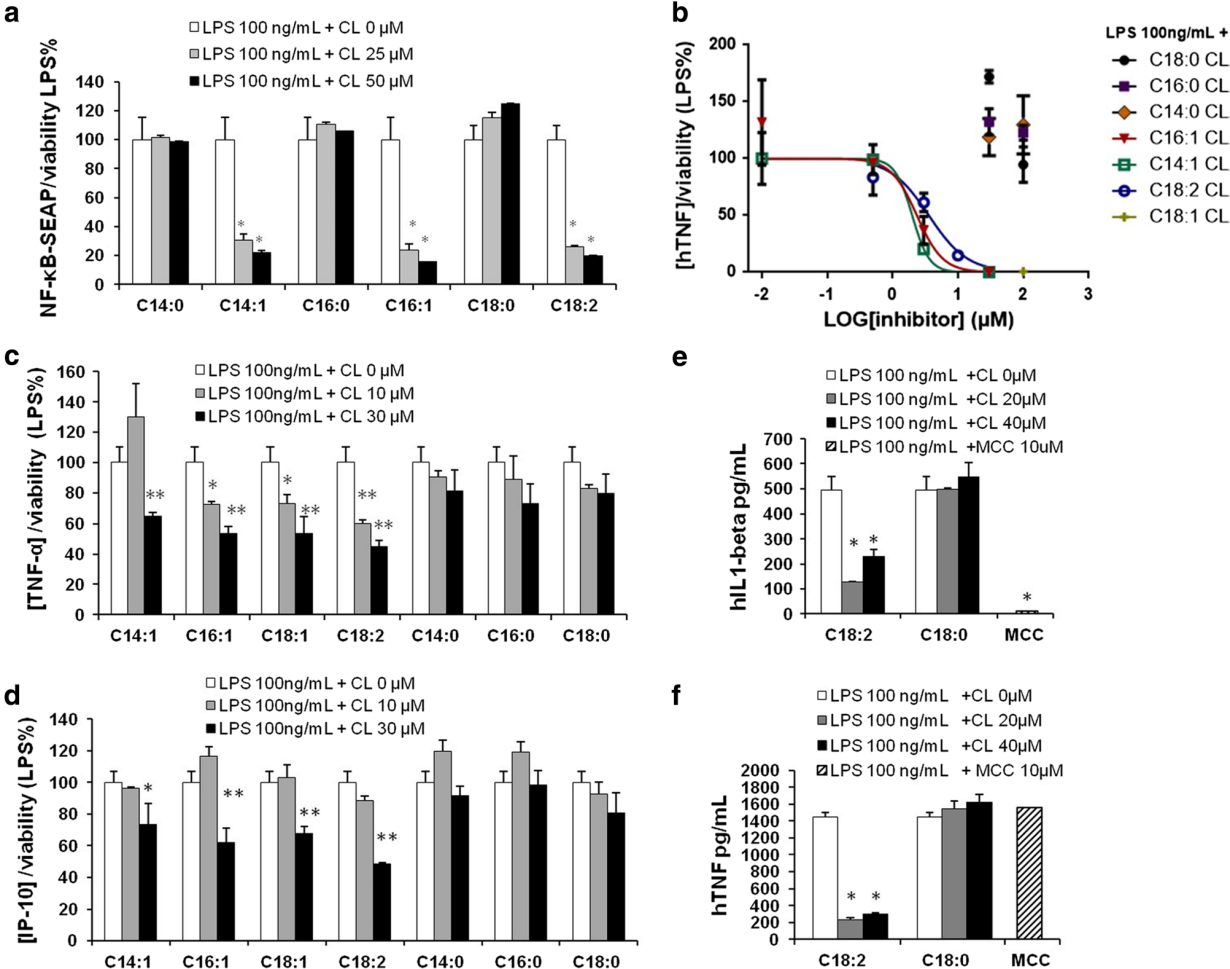
Unsaturated but not saturated cardiolipins inhibit 100ng/mL LPS dependent pro-inflammatory response (**a**) Murine macrophages RAW-Blue™ cells were incubated 16 hours with LPS 100ng/mL alone (+ CL 0 μM) or together with the indicated amount of each CL, NF-κB activation was quantified in collected supernatants via Quanti-Blue test, normalized on cell viability measured via MTT test and reported here as the percentage of the values measured for LPS in absence of CL (4 fold induction compared to the unstimulated condition). (**b**) Primed THP1 cells were incubated 1 h with the indicated amount of each CL, washed and incubated 5 hours with 100 ng/mL of LPS. TNF-α was quantified in collected supernatants by ELISA assays. (**c**, **d**) Primary BMDMs were incubated 16 h with LPS 100ng/mL alone or together with the indicated amount of each CL. TNF-α and IP-10 were quantified in collected supernatants by ELISA assays, normalized on cell viability measured via MTT test and reported here as the percentage of the values measured for LPS in absence of CL (2056 pg/mL of TNF-α, 6000 pg/mL of IP-10). (**e**, **f**) Human PBMCs were incubated for 4 h (**f**) or 16 h (**e**) with LPS 100ng/mL alone or together with the indicated amount of each CL or the NLRP3 inhibitor MCC950. TNF-α and IL-1β were quantified in collected supernatants by ELISA assays. Graphs are representative of experiments from 5 different donors. Each bar represents the mean + standard deviation of three biological replicates (n = 3). Graphs are representative of at least three independent experiments. Unpaired t-test: decrease compared to LPS is not significant if p>0,05 (no symbol); *p ≤ 0.002 (**a**, **e**, **f**) *p≤ 0.05, **p ≤ 0.021 (**c**, **d**)

Bovine heart and *E. coli* CLs were previously shown to inhibit LPS-induced cytokine secretion [11]. To identify the role of chain length or saturation in such anti-inflammatory properties, we investigated the ability of unsaturated and saturated C14, C16, and C18 CLs (Fig. 1 and S1) to inhibit LPS-dependent NF-κB activation and cytokine secretion in human and murine cells. We performed a set of screening experiments with the RAW and PMA-primed THP-1 immortalized macrophages which closely mimic the responses of primary mouse and human macrophages, respectively [22, 23]. Immortalized murine macrophages RAW-Blue™ were co-incubated with CLs and LPS for 16 h. NF-κB activation in RAW-Blue cells was quantified by the measurement of NF-κB-dependent SEAP production in the supernatant, while cell viability was evaluated by MTT test (Fig. S5b). As shown in Fig. 2, unsaturated but not saturated CLs were able to inhibit, in a dose-dependent manner, LPS-induced NF-κB activation in RAW-Blue™ cells (Fig. 2a). To estimate the dose-dependence of CL anti-inflammatory effects in human cells, we pre-incubated PMA-primed THP-1 cells with CLs for 1 h, and then, cells were washed to remove unbounded CL from the media before adding LPS. After 5 h, supernatants were collected and TNF-α quantified in the supernatants as a measure of TLR4/NF-κB activation, while cell viability was evaluated by MTT test (Fig. S7b). The timing of incubation was shortened to 5 h as the response to LPS was the highest at that timing. As shown in Fig. 2b, unsaturated but not saturated CLs were able to inhibit, in a dose-dependent manner, LPS-induced TNF-α secretion in primed THP-1 cells (Fig. 2b).

To validate our results, we used primary macrophages derived from murine bone marrow (BMDMs). BMDMs were co-incubated for 16 h with CL and LPS and TNF-α and IP-10 secretion was quantified in cell supernatants (Fig. 2c, d), while cell viability was assessed by MTT test (Fig. S6c). As shown in Fig. 2c, d, unsaturated but not saturated CLs were able to inhibit, in a dose-dependent manner, LPS-induced cytokine secretion in BMDMs.

MTT results showed a decrease of viability when Raw, THP1, and BMDMs cells were co-incubated with LPS and saturated CLs (Figs. S5b, S6c, and S7b). This was reflected in an apparent inhibition of LPS-induced inflammatory response (Figs. S5a, S6a, b, and S7a). In Fig. 2, data were normalized to cell viability measured by MTT test to exclude the contribution of toxicity to the decrease of cell response. When data are normalized to viability (Fig. 2), the decreases induced by saturated CLs disappear, meaning that, per each viable cell, no inhibitory effect of saturated CLs was occurring.

In contrast to macrophages, activation of TLR4 by LPS in human monocytes leads also to the activation of the so-called alternative NLRP3 inflammasome pathway and IL-1β secretion [24]. To study the ability of cardiolipin to inhibit this pathway, peripheral blood mononuclear cells from human donors (PBMCs), which include monocytes, were co-incubated with CLs and LPS. Supernatants were collected after 4 h to assess TNF-α and 16 h to assess IL-1β secretion, as this was the timing at which we observed the highest response to LPS. In PBMCs, LPS alone was able to induce the release of IL-1β, suggesting that the alternative NLRP3 inflammasome pathway was activated (Fig. 2e). We then tested the ability of CLs to inhibit LPS-dependent inflammatory IL-1β release in human PBMCs. The inhibitory activity of CL in PBMCs was compared to the inhibitor MCC950 that is a specific NLRP3 inhibitor [25]. As expected, MCC950 was able to inhibit IL-1β secretion but not TNF-α, whereas unsaturated CL inhibited both cytokines (Fig. 2e, f), suggesting that the inhibition of the alternative inflammasome pathway occurs at the TLR4 level. We also confirmed that the use of saturated CLs had no inhibitory effects. The relevance of human blood cells to our study is also that CL is a component of human plasma lipoprotein; it is found in blood from haemodialysis patients and that blood cells from Barth Syndrome patients present modified CLs [26–29].

### Saturated CLs induce pro-inflammatory cytokine secretion through TLR4 activation

We performed preliminary experiments to evaluate the pro-inflammatory properties of CLs by incubating CL liposomes with two different macrophage cell lines, RAW-Blue and immortalized BMDMs, which closely mimic the responses of primary mouse macrophages [20, 23].

**Fig. 3:**
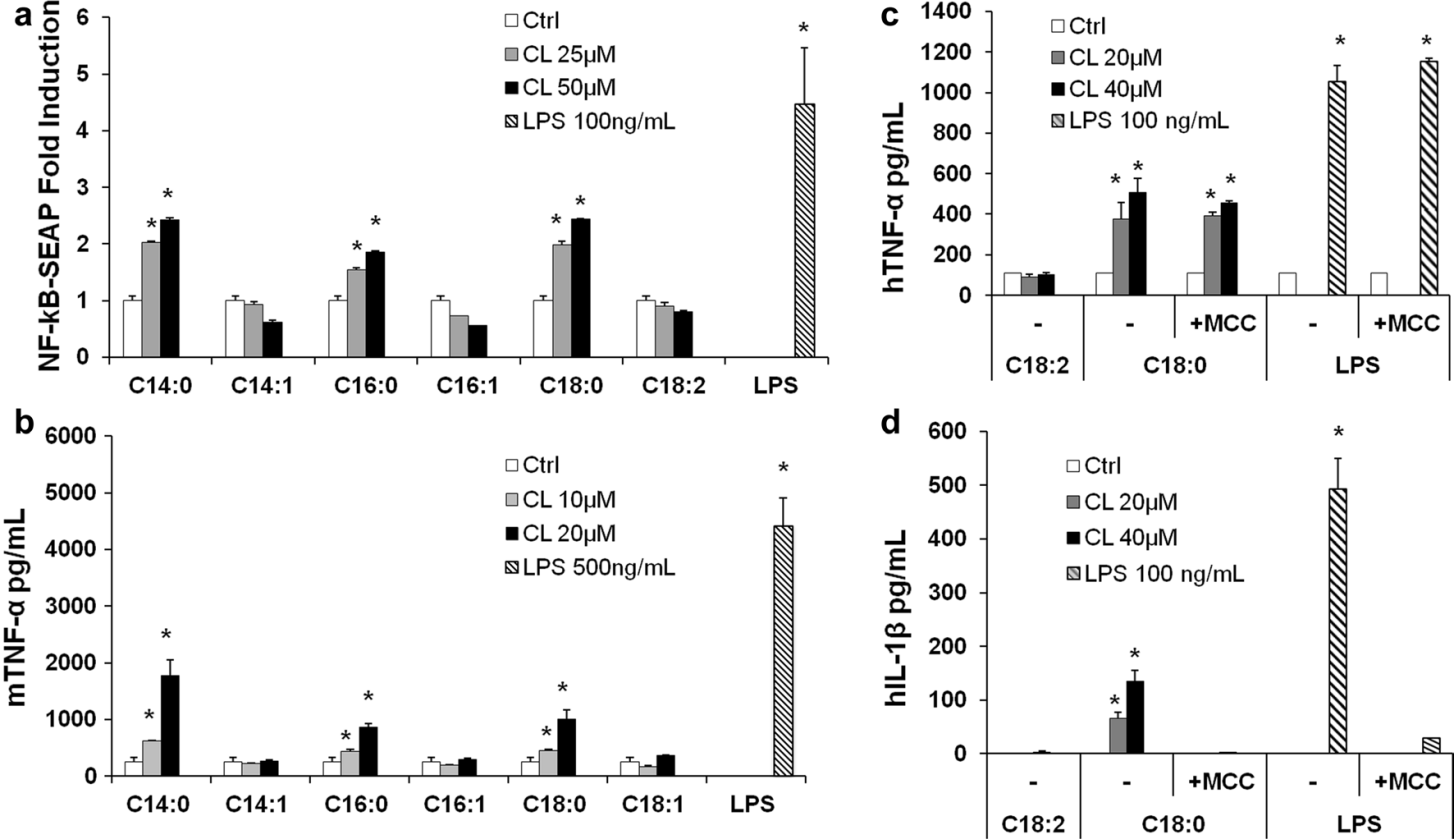
Saturated but not unsaturated CLs have pro-inflammatory properties. **a** RAW-Blue™ cells were incubated overnight with the indicated amount of CL or LPS, supernatants were collected, and NF-κB activation quantified via Quanti-Blue™ test and reported here as fold induction as compared to control. Unpaired *t* test: differences with respect to the control (Ctrl) are not significant if *p* > 0.05 (no symbol); *p ≤ 0.0007. **b** Immortalized BMDM cells were incubated overnight with the indicated amount of CL or LPS; supernatants were collected and TNF-α quantified via ELISA assay. **c**, **d** Primary human monocytes were incubated overnight with the indicated amount of CL or LPS with or without the NLRP3 inhibitor MCC950, supernatants were collected, and TNF-α and IL-1β were quantified via ELISA assay. Graphs are representative of experiments from six different donors. Unpaired *t* test: differences with respect to the control (Ctrl) are not significant if *p* > 0.05 (no symbol); *p ≤ 0.021. Each bar represents the mean + standard deviation of three biological replicates (*n* = 3). Graphs are representative of at least three independent experiments

In murine macrophages C14:0, C16:0, and C18:0 CLs but not C14:1, C16:1, C18:1, or C18:2 CLs induced NF-κB activation and TNF-α secretion (Fig. 3a, b). We then validated the results in primary BMDMs, where, as shown in the supplemental Fig. S2, saturated CL induced TNF-α and IP-10 secretion.

C18:0 and C18:2 CLs’ pro-inflammatory activities were validated in primary monocytes isolated from human donor blood. C18:0 CL but not C18:2 CL reproduced LPS activity inducing TNF-α and IL-1β secretion in a dose-dependent manner (Fig. 3c, d). IL-1β secretion was inhibited by the NLRP3 inhibitor MCC950, which demonstrates the activation of the alternative NLRP3 inflammasome pathway by saturated CL in human monocytes (Fig. 3d).

**Fig. 4:**
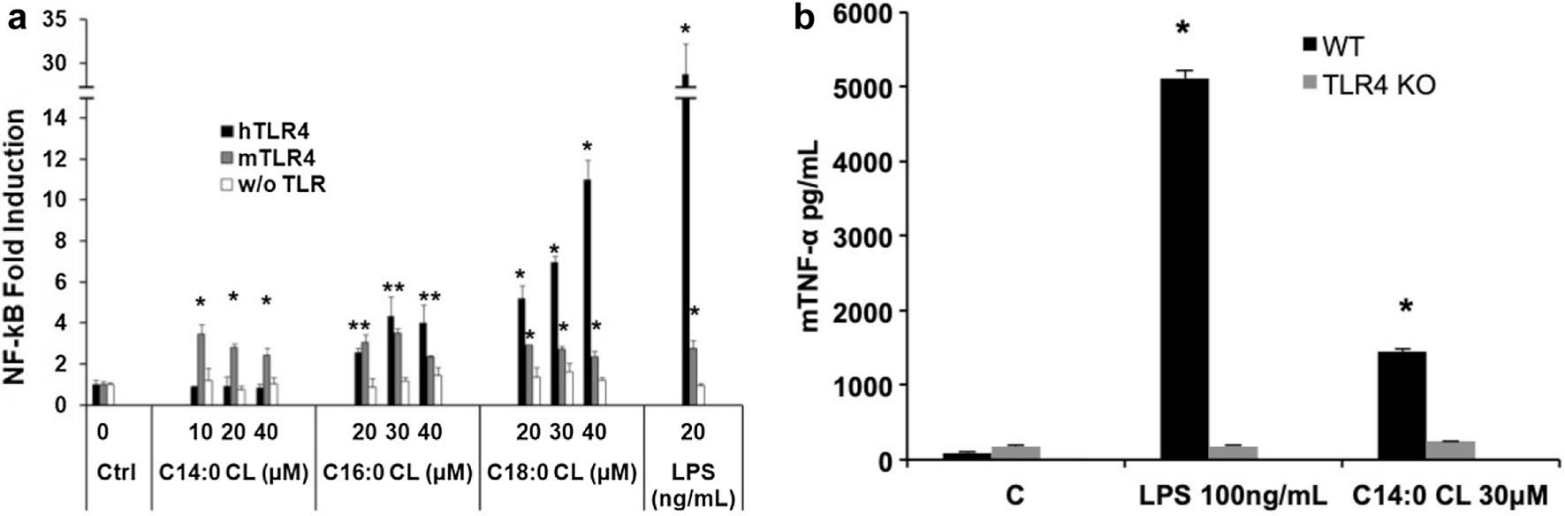
Saturated CLs are TLR4 agonists. **a** HEK293 cells were transfected with plasmids encoding murine TLR4, MD2, and CD14 (mTLR4), human TLR4, MD2, and CD14 (hTLR4) or with empty pcDNA3.1 plasmid, MD2 and CD14 (w/o TLR) together with a luciferase reporter plasmid dependent on NF-κB activation and a constitutively active reporter vector-encoding renilla luciferase. Two days after transfection, cells were incubated for 6 h with medium alone (Ctrl) or the indicated amount of CLs or LPS. Luciferase and renilla were quantified in cell lysate, normalized and reported here as fold induction as compared to control. Unpaired *t* test: differences compared to the control are not significant if *p* > 0.05(no symbol); *p ≤ 0.005. **b** WT and TLR4-KO iBMDM cells were incubated overnight with medium (**c**), LPS or C14:0 CL. For each condition, mTNF-α was measured by ELISA assay. Unpaired *t* test: differences with respect to the control are not significant if *p* > 0.05(no symbol); *p ≤ 0.0001. Each bar represents the mean + standard deviation of three biological replicates (*n* = 3). Graphs are representative of at least three independent experiments

The first signaling event of these pathways is NF-κB activation through TLR4 activation [24]. To investigate how the individual CD14, TLR4, and MD2 proteins contribute to the responses of cells to CL, we used the HEK transfection model where we can control which protein (including from different species) may be added to the cells and their contribution to CL activation of Nf-κB [21, 30, 31]. We transfected HEK293 cells with human or murine CD14 and MD2 with or without TLR4 and incubated them with saturated CLs for 6 h (Fig. 4a). All three saturated CLs activated murine TLR4. By contrast, only C16:0 and C18:0 activated the human receptor. To further confirm the role of TLR4, we compared TNF-α secretion induced by C14:0 CL in wild-type (WT) or TLR4 knock out (TLR4 KO) immortalized BMDMs. Both LPS and C14:0 CL induced TNF-α secretion in WT but not in TLR4-KO BMDMs (Fig. 4b). TLR4-KO BMDMs were normally responsive to TLR2 ligand Pam_3_CSK_4_ (data not shown).

### Activation of TLR4 by cardiolipin requires CD14 and MD2

**Fig. 5:**
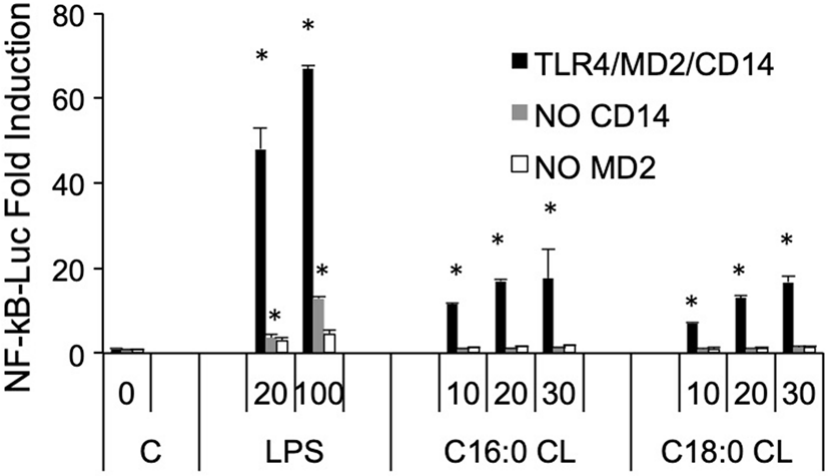
TLR4 activation by saturated CLs requires MD2 and CD14. HEK293 cells were transfected with plasmids encoding hTLR4, hMD2, and hCD14 (black bars), hTLR4 and hMD2 (gray bars), or hTLR4 and hCD14 (white bars) together with a luciferase reporter plasmid dependent on NF-κB activation and a constitutively active reporter vector-encoding renilla luciferase. Two days after transfection, cells were incubated for 6 h with medium alone (C, 0) or the indicated amount of CLs (μM) or LPS (ng/mL). Luciferase and renilla were quantified in cell lysate, and normalized and reported here as fold induction as compared to control. Unpaired *t* test: differences compared to the control (C) are not significant if *p* > 0.05(no symbol); *p ≤ 0.02. Each bar represents the mean + standard deviation of three biological replicates (*n* = 3). Graphs are representative of at least three independent experiments

Next, we investigated whether CD14 and MD2 are required for inflammatory signaling induced by saturated CLs. NF-κB was not induced by C16:0 or C18:0 CLs in HEK293 cells transfected with TLR4 in the absence of CD14 or MD2, demonstrating that, like LPS, both co-receptors are essential for TLR4 activation by saturated CLs. Residual activity of LPS in cells transfected without CD14 is likely due to the presence of soluble CD14 in serum [32, 33] (Fig. 5).

### Unsaturated and C14:0 CLs are competitive human TLR4 antagonists

**Fig. 6:**
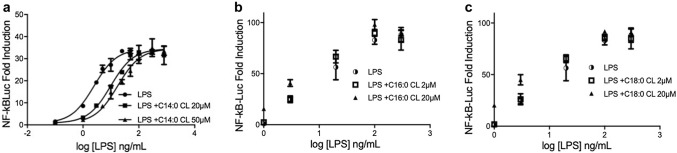
C14:0 is a competitive human TLR4 antagonist. HEK293 cells were transfected with plasmids encoding hTLR4, hMD2, and hCD14 together with a luciferase reporter plasmid dependent on NF-κB activation and a constitutively active reporter vector-encoding renilla luciferase. Two days after transfection, cells were incubated for 1 h with medium alone or the indicated amount of CLs, then washed and incubated 6 h with increasing amount of LPS. Luciferase and renilla were then quantified in cell lysate, and normalized and reported here as fold induction as compared to control. Each bar represents the mean ± standard deviation of three biological replicates (*n* = 3). Graphs are representative of at least three independent experiments

In contrast to C16:0 and C18:0, C14:0 CL acted as an agonist in murine but not human cells (Fig. 3), a property similar to that of the LPS variant lipid IVa [34]. to establish whether C14:0, like lipid IVa, is a weak human TLR4 antagonist, we tested the ability of all saturated CLs to inhibit signaling induced by LPS over a range of concentrations. For each CL, cells transfected with human TLR4/MD2/CD14 were pre-incubated with medium alone or two different CL concentrations for 1 h, and then washed and incubated for 6 h with increasing amounts of LPS (from 0 to 1000 ng/mL). As shown in Fig. 6, the NF-κB activation induced by low LPS concentrations (< 100 ng/mL) was decreased in cells pre-incubated with C14:0 CL. The dose–response curve of LPS was shifted to the right. This indicates that C14:0 CL acts as a competitive inhibitor of LPS-induced signaling in human cells by occupying LPS-binding site at the TLR4/MD2 interface [35, 36]. As expected, the agonists C16:0 and C18:0 CL did not inhibit any LPS concentration. Furthermore, all saturated CLs did not inhibit lower LPS concentration in assays using murine RAW cells (Fig. S3).

**Fig. 7:**
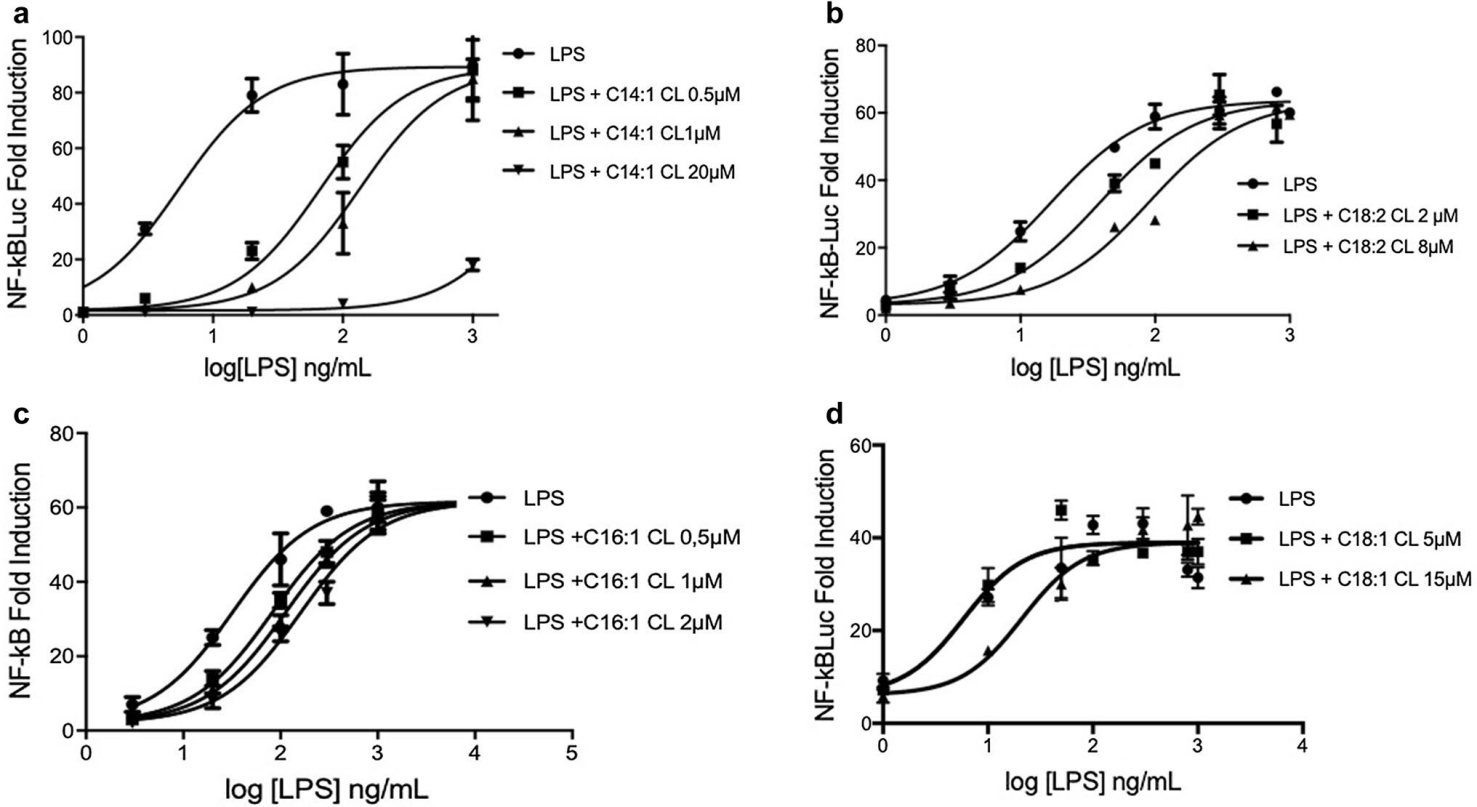
Unsaturated cardiolipins are competitive human TLR4 antagonists. HEK293 cells were transfected with plasmids encoding hTLR4, hMD2, and hCD14 together with a luciferase reporter plasmid dependent on NF-κB activation and a constitutively active reporter vector-encoding renilla luciferase. Two days after transfection, cells were incubated for 1 h with medium alone or the indicated amount of CLs, and then washed and incubated 6 h with increasing amount of LPS. Luciferase and renilla were then quantified in cell lysate, and normalized and reported here as fold induction as compared to control. Each bar represents the mean ± standard deviation of three biological replicates (*n* = 3). Graphs are representative of at least three independent experiments

We next tested whether, like C14:0, unsaturated CLs are competitive inhibitors of LPS mediated signaling. As shown in Fig. 7, all unsaturated CLs shifted the dose–response curves to the right without affecting the maximal level of signaling, the hallmark of competitive antagonists.

### The potency of anti-inflammatory CLs depends on chain length and the degree of unsaturation

**Fig. 8:**
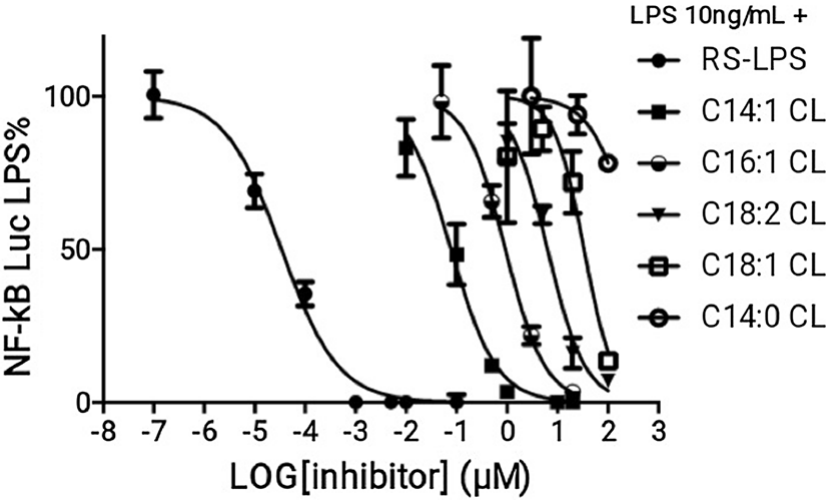
Shorter and more unsaturated CLs are more potent human TLR4 antagonists. HEK293 cells were transfected with plasmids encoding hTLR4, hMD2, and hCD14 together with a luciferase reporter plasmid dependent on NF-κB activation and a constitutively active reporter vector-encoding renilla luciferase. Two days after transfection, cells were incubated for 1 h with increasing amount of CLs, and then washed and incubated 6 h with the indicated amount of LPS. Luciferase and Renilla were then quantified in cell lysate, and normalized and reported here as percentage of the value measured for LPS alone. Each bar represents the mean ± standard deviation of three biological replicates (*n* = 3). Graphs are representative of at least three independent experiments. For C14:0, it was only possible to plot three points due to problems of solubility and cell viability

To quantify and compare the potency of CLs as inhibitors, we determined IC_50_ values in HEK293 cells transfected with human TLR4/MD2/CD14, for each of the antagonists (Fig. 8 and Table 1). The most potent CL was C14:1 (IC_50_ = 77 nM) and the weakest C14:0 (300 µM approx.). All CLs were less potent than LPS derived from *Rhodobacter sphaeroides* (RS-LPS) with a measured IC_50_ of 34 pM (Table 1), consistent with the published values [37]. These data show that shorter chain length and higher levels of unsaturation increase potency with a 400-fold difference between C14:1 and C18:1 and a sixfold difference between C18:2 and C18:1 (Table 1).

**Table 1:** IC_50_ of CLs and RS-LPS calculated from data in Fig. 8 using Prism fitting analysis

Antagonist	RS-LPS	C14:1 CL	C16:1	C18:2 CL	C18:1 CL	C14:0 CL
IC_50_ (µM)	3.402e−5	0.07785	0.9249	6.347	33.53	324.8*

*Estimated value

## Discussion

Consistent with our results, three studies have previously shown that heart CL inhibits LPS-induced pro-inflammatory cytokines secretion [11–13]. Mueller and co-workers attributed the anti-inflammatory properties to the inhibition of LBP, but did not investigate CD14 or TLR4 binding, which could occur subsequently. Coats and co-workers studied mainly fecal lipid extract and suggested an interesting role of CL in intestinal homeostasis, without elucidating the mechanism of TLR4 inhibition. Finally, Balasubramanian and co-workers demonstrated that extracellular bovine heart CL was able to inhibit LPS-dependent pro-inflammatory cytokines secretion in immortalized and primary human and murine macrophages. Although they highlighted the anti-inflammatory properties of extracellular CL and showed that CL competes with LPS for binding recombinant immobilized MD2, these studies did not investigate the ability of CL to interfere with the binding of LPS to TLR4/MD2 complex or with the TLR4 dimerization. Our analysis of competition between LPS and CLs (Figs. 6 and 7) shows that C14:0, heart CL (C18:2), and the three synthetic unsaturated CLs (C14:1, C16:1 and C18:1) are competitive TLR4 antagonists that inhibit LPS signaling by occupying its binding pocket in human TLR4/MD2 complex in living cells.

Furthermore, we have shown that unsaturated CLs regardless of their chain length are able to inhibit LPS-dependent inflammatory cytokine secretion and the activation of the alternative NLRP3 inflammasome pathway (Figs. 2 and 7), while cardiolipins with saturated chains longer than 14 carbon atoms, like those found in patients with BTHS and in some bacteria [2, 38, 39], lose their anti-inflammatory properties (Fig. 2), and are instead able to mimic LPS by activating TLR4 and inducing pro-inflammatory cytokine secretion in macrophages and human blood-derived monocytes (Figs. 3, 4, and 5). Where the acyl chain length is more than 14 saturated CLs always activate, whereas unsaturated CLs inhibit TLR4. At human TLR4, saturated C14 CL acts as a very weak antagonist with an IC_50_ > 328 uM. The possibility that TLR4 signaling is caused by contaminating lipids is overcome in this study by the use of synthetic CLs.

The role of TLR4 in promoting maturation of antigen presenting cells and directing the induction of adaptive immune responses makes TLR4 agonists good vaccine adjuvants for use in infectious diseases or cancer [40, 41]. On the other hand, the emerging role of TLR4 and NLRP3 activation in inflammatory and autoimmune diseases, such as sepsis and lupus, among others has suggested the use of TLR4 and NLRP3 antagonists in therapy [41–45]. Our data extend the library of TLR4 and alternative NLRP3 inhibitors and activators to cardiolipins, and also provide a new rationale for the synthesis of modulators with lower price and higher biocompatibility than LPS derivatives. Indeed, our work contributes to elucidating the minimal structural requirement for TLR4 recognition, showing that, in addition to the polysaccharide moiety of the LPS inner core [46], the saccharide moieties of lipid A, considered as the minimal structure for the LPS activity, are also not necessary for TLR4 activation and inhibition.

A role of unsaturated acyl chains in TLR4 inhibition was suggested by their presence in natural and synthetic TLR4 antagonists [17, 47], although those molecules differ from TLR4 agonists also in terms of linker, number, and length of acyl chains. In this study, by comparing synthetic molecules differing only in terms of unsaturation degree (Fig. 1), we formally demonstrated that double bonds discriminate between TLR4 antagonistic and agonistic activity, with the exception of molecules with saturated acyl chains shorter than 14 carbon atoms, which are still murine TLR4 agonist but weak human TLR4 antagonist. Moreover, tetra-acylated lipids with a shorter chain length and a higher number of unsaturated bonds are more potent antagonists at human TLR4, which is an important consideration for the synthesis of new human TLR4 antagonists.

The species-specific properties of the saturated C14:0 CL are reminiscent of lipid IVa, an underacylated form of LPS, and some synthetic saturated LPS derivatives that activate mouse TLR4/MD2, but are weak antagonists in human [34, 48]. In human TLR4/MD2, lipid IVa adopts a conformation in the MD2-binding pocket that prevents the hydrophobic interface and ionic contacts with a second TLR4/MD2 heterodimer required for the assembly of an active hetero-tetramer [49]. By contrast in mouse subtle differences in MD2 allow the lipid IVa molecule to re-orientate, rotating through 120° in the MD2-binding pocket which allows these critical contacts to form [34, 50].

Murine and human TLR4 antagonists Lipid A from *R. sphaeroides* (RS-LPS) and Eritoran have a single double bond that causes a kink in the acyl chain. This affects the way that the molecules can pack into the hydrophobic core of the MD2 co-receptor, locking it into a conformation buried in the MD2 pocket and unable to make the interaction required for TLR4 activation, but, nevertheless, preventing agonist LPS binding [19, 49]. It is likely that unsaturated CLs have a similar mechanism of action, whereas saturated CL can mimic agonist lipid A conformation and contact key residues for TLR4 dimerization (see supplemental Fig. S4 for a graphical illustration). However, the precise configuration adopted by unsaturated CLs in the MD2-binding pocket, and the way that this differs from that of the saturated CLs will require structural studies.

Cardiolipin is normally located in the inner mitochondrial membrane and is externalized after mitochondrial damage induced by LPS stimulation [51]. Similar to bacteria, mitochondria that expose CL at their surface are phagocytised by macrophages [11]. Therefore, CL might be sensed by TLR4 at the surface of intact cells when damaged mitochondria and/or mitochondria-derived vesicles are secreted into the extracellular environment [52]. A similar mechanism of detection has been described for mitochondrial DNA that activates TLR9 [52]. The need of CD14 in CL signaling demonstrated in this study (Fig. 5) strongly suggests that CD14 may transfer CL from mitochondrial membranes to TLR4. Indeed, CD14 is necessary to help LPS extraction from bacterial membranes and favor TLR4 presentation [53]. Therefore, endogenous CL might have a role in down-regulating inflammation during bacterially induced cell damage. In BTHS, a genetic modification of the enzyme tafazzin causes a decrease of unsaturated CLs and an increase of saturated ones. Our results suggest that, in patients affected by this disease, the negative regulation of the inflammation would be impaired and the exposed saturated CLs would act instead as pro-inflammatory DAMPs, explaining the chronic inflammation associated with the disease.

Recently, the anti-inflammatory activity of CL has been investigated in a mouse model of lung inflammation [54]. Mice treated with LPS followed by cardiolipin had higher levels of pro-inflammatory cytokines but lower levels of the anti inflammatory cytokine IL-10. This suggests that CLs can modify LPS signaling in vivo. In contrast to our results and contributions from others [11–13], CL was administered after LPS. The known ability of the dimeric TLR4/MD2/LPS complex to internalize and signal from the endosomes might prevent CL binding to TLR4 and LPS inhibition if LPS has already been administered. Therefore, the work of Chakraborty and co-workers does not rule out an anti-inflammatory role of CL in vivo.

A link between CL and TLR-immunity in vivo is also suggested by its association with diseases characterized by an inflammatory state such as BTHS, Antiphospholipid Syndrome, inflammatory bowel diseases, and Parkinson’s [39, 55–57]. Furthermore, antibodies against CL have been identified in patients with lupus, inflammatory bowel disease, and Antiphospholipid Syndrome [55, 56, 58], diseases that are thought to be linked to TLR4 [59–62]. Our work suggests that investigating CL modifications and its interaction with TLR4 in patients affected by these diseases may provide new understanding of their pathophysiology.

## Conclusions

This work demonstrates that mammalian cardiolipins inhibit inflammation induced by bacterial LPS by blocking its interaction with the immune receptor TLR4 and that a minor structural modification, consisting of the elimination of double bonds from its acyl chains, transforms cardiolipin into an activator of TLR4 that, instead of inhibiting, induces inflammation as bacterial LPS. This has important implications on the synthesis of new modulators of TLR4 to be used as vaccine adjuvants or therapeutics and for understanding the immune response to bacteria as well as the pathology of Barth syndrome both of which involve saturated cardiolipins.

## Electronic supplementary material

Below is the link to the electronic supplementary material.

Supplementary material 1 (PDF 695 kb)

## References

[1] Houtkooper RH, Rodenburg RJ, Thiels C (2009). Cardiolipin and monolysocardiolipin analysis in fibroblasts, lymphocytes, and tissues using high-performance liquid chromatography–mass spectrometry as a diagnostic test for Barth syndrome. Anal Biochem.

[2] Oemer G, Lackner K, Muigg K (2018). Molecular structural diversity of mitochondrial cardiolipins. Proc Natl Acad Sci USA.

[3] Zhou Y, Peisker H, Dörmann P (2016). Molecular species composition of plant cardiolipin determined by liquid chromatography mass spectrometry. J Lipid Res.

[4] Schlame M, Brody S, Hostetler KY (1993). Mitochondrial cardiolipin in diverse eukaryotes. Eur J Biochem.

[5] Mileykovskaya E, Dowhan W (2014). Cardiolipin-dependent formation of mitochondrial respiratory supercomplexes. Chem Phys Lipids.

[6] Ren M, Phoon CKL, Schlame M (2014). Metabolism and function of mitochondrial cardiolipin. Prog Lipid Res.

[7] Maguire JJ, Tyurina YY, Mohammadyani D (2017). Known unknowns of cardiolipin signaling: the best is yet to come. Biochim Biophys Acta.

[8] Ikon N, Ryan RO (2017). Barth syndrome: connecting cardiolipin to cardiomyopathy. Lipids.

[9] van Raam B, Kuijpers T (2009). Mitochondrial defects lie at the basis of neutropenia in Barth syndrome. Curr Opin Hematol.

[10] Wilson LD, Al-Majid S, Rakovski CS, Md CDS (2012). Higher IL-6 and IL6:IGF ratio in patients with barth syndrome. J Inflamm (Lond).

[11] Balasubramanian K, Maeda A, Lee JS (2015). Dichotomous roles for externalized cardiolipin in extracellular signaling: promotion of phagocytosis and attenuation of innate immunity. Sci Signal.

[12] Coats SR, Hashim A, Paramonov NA (2016). Cardiolipins act as a selective barrier to toll-like receptor 4 activation in the intestine. Appl Environ Microbiol.

[13] Mueller M, Brandenburg K, Dedrick R (2005). Phospholipids inhibit lipopolysaccharide (LPS)-induced cell activation: a role for LPS-binding protein. J Immunol.

[14] Gay NJ, Symmons MF, Gangloff M, Bryant CE (2014). Assembly and localization of Toll-like receptor signalling complexes. Nat Rev Immunol.

[15] Kagan JC, Barton GM (2014). Emerging principles governing signal transduction by pattern-recognition receptors. Cold Spring Harb Perspect Biol.

[16] Latty SL, Sakai J, Hopkins L (2018). Activation of Toll-like receptors nucleates assembly of the MyDDosome signaling hub. eLife.

[17] Bryant CE, Spring DR, Gangloff M, Gay NJ (2010). The molecular basis of the host response to lipopolysaccharide. Nat Rev Microbiol.

[18] Erridge C, Bennett-Guerrero E, Poxton IR (2002). Structure and function of lipopolysaccharides. Microbes Infect.

[19] Kim HM, Park BS, Kim J-I (2007). Crystal structure of the TLR4-MD-2 complex with bound endotoxin antagonist Eritoran. Cell.

[20] Harris J, Hartman M, Roche C (2011). Autophagy controls IL-1β secretion by targeting Pro-IL-1β for degradation. J Biol Chem.

[21] Pizzuto M, Gangloff M, Scherman D (2016). Toll-like receptor 2 promiscuity is responsible for the immunostimulatory activity of nucleic acid nanocarriers. J Control Release.

[22] Starr T, Bauler TJ, Malik-Kale P, Steele-Mortimer O (2018). The phorbol 12-myristate-13-acetate differentiation protocol is critical to the interaction of THP-1 macrophages with *Salmonella typhimurium*. PLoS One.

[23] Berghaus LJ, Moore JN, Hurley DJ (2010). Innate immune responses of primary murine macrophage-lineage cells and RAW 264.7 cells to ligands of Toll-like receptors 2, 3, and 4. Comp Immunol Microbiol Infect Dis.

[24] Gaidt MM, Ebert TS, Chauhan D (2016). Human monocytes engage an alternative inflammasome pathway. Immunity.

[25] Coll RC, Robertson AAB, Chae JJ (2015). A small-molecule inhibitor of the NLRP3 inflammasome for the treatment of inflammatory diseases. Nat Med.

[26] Valianpour F, Wanders RJA, Barth PG (2002). Quantitative and compositional study of cardiolipin in platelets by electrospray ionization mass spectrometry: application for the identification of Barth syndrome patients. Clin Chem.

[27] Kulik W, van Lenthe H, Stet FS (2008). Bloodspot assay using HPLC–Tandem mass spectrometry for detection of Barth syndrome. Clin Chem.

[28] Deguchi H, Fernández JA, Hackeng TM (2000). Cardiolipin is a normal component of human plasma lipoproteins. PNAS.

[29] Antonopoulou S, Demopoulos CA, Iatrou C (1996). Blood cardiolipin in haemodialysis patients. Its implication in the biological action of platelet-activating factor. Int J Biochem Cell Biol.

[30] Tandon A, Harioudh MK, Ishrat N (2018). An MD2-derived peptide promotes LPS aggregation, facilitates its internalization in THP-1 cells, and inhibits LPS-induced pro-inflammatory responses. Cell Mol Life Sci.

[31] Lonez C, Irvine KL, Pizzuto M (2015). Critical residues involved in Toll-like receptor 4 activation by cationic lipid nanocarriers are not located at the lipopolysaccharide-binding interface. Cell Mol Life Sci.

[32] Kielian TL, Blecha F (1995). CD14 and other recognition molecules for lipopolysaccharide: a review. Immunopharmacology.

[33] Yang Z, Breider MA, Carroll RC (1996). Soluble CD14 and lipopolysaccharide-binding protein from bovine serum enable bacterial lipopolysaccharide-mediated cytotoxicity and activation of bovine vascular endothelial cells in vitro. J Leukoc Biol.

[34] Walsh C, Gangloff M, Monie T (2008). Elucidation of the MD-2/TLR4 interface required for signaling by lipid IVa. J Immunol.

[35] Lazareno S, Birdsall NJ (1993). Estimation of competitive antagonist affinity from functional inhibition curves using the Gaddum, Schild and Cheng-Prusoff equations. Br J Pharmacol.

[36] Schild HO (1957). Drug antagonism and pAx. Pharmacol Rev.

[37] Rose JR, Christ WJ, Bristol JR (1995). Agonistic and antagonistic activities of bacterially derived *Rhodobacter sphaeroides* lipid A: comparison with activities of synthetic material of the proposed structure and analogs. Infect Immun.

[38] Schlame M (2013). Cardiolipin remodeling and the function of tafazzin. Biochimica et Biophysica Acta.

[39] Schlame M, Ren M (2006). Barth syndrome, a human disorder of cardiolipin metabolism. FEBS Lett.

[40] Dowling JK, Mansell A (2016). Toll-like receptors: the swiss army knife of immunity and vaccine development. Clin Transl Immunol.

[41] O’Neill LAJ, Bryant CE, Doyle SL (2009). Therapeutic targeting of Toll-like receptors for infectious and inflammatory diseases and cancer. Pharmacol Rev.

[42] de Torre-Minguela C, Mesa del Castillo P, Pelegrín P (2017). The NLRP3 and pyrin inflammasomes: implications in the pathophysiology of autoinflammatory diseases. Front Immunol.

[43] Guo H, Callaway JB, Ting JP-Y (2015). Inflammasomes: mechanism of action, role in disease, and therapeutics. Nat Med.

[44] Peri F, Piazza P (2012). Therapeutic targeting of innate immunity with Toll-like receptor 4 (TLR4) antagonists. Biotechnol Adv.

[45] Wang L, Wang F-S, Gershwin ME (2015). Human autoimmune diseases: a comprehensive update. J Intern Med.

[46] Cochet F, Peri F (2017). The role of carbohydrates in the lipopolysaccharide (LPS)/Toll-like receptor 4 (TLR4) signalling. Int J Mol Sci.

[47] Peri F, Calabrese V (2014). Toll-like receptor 4 (TLR4) modulation by synthetic and natural compounds: an update. J Med Chem.

[48] Bazin HG, Murray TJ, Bowen WS (2008). The ‘Ethereal’ nature of TLR4 agonism and antagonism in the AGP class of lipid A mimetics. Bioorg Med Chem Lett.

[49] Meng J, Lien E, Golenbock DT (2010). MD-2-mediated ionic interactions between lipid A and TLR4 are essential for receptor activation. J Biol Chem.

[50] Ohto U, Fukase K, Miyake K, Shimizu T (2012). Structural basis of species-specific endotoxin sensing by innate immune receptor TLR4/MD-2. Proc Natl Acad Sci USA.

[51] Elliott EI, Miller AN, Banoth B (2018). Cutting edge: mitochondrial assembly of the NLRP3 inflammasome complex is initiated at priming. J Immunol.

[52] West AP, Shadel GS (2017). Mitochondrial DNA in innate immune responses and inflammatory pathology. Nat Rev Immunol.

[53] Ostuni R, Zanoni I, Granucci F (2010). Deciphering the complexity of Toll-like receptor signaling. Cell Mol Life Sci.

[54] Chakraborty K, Raundhal M, Chen BB (2017). The mito-DAMP cardiolipin blocks IL-10 production causing persistent inflammation during bacterial pneumonia. Nat Commun.

[55] Aichbichler BW, Petritsch W, Reicht GA (1999). Anti-cardiolipin antibodies in patients with inflammatory bowel disease. Dig Dis Sci.

[56] Rauch J, Dieudé M, Subang R, Levine J (2010). The dual role of innate immunity in the antiphospholipid syndrome. Lupus.

[57] Tufekci KU, Meuwissen R, Genc S, Genc K (2012). Inflammation in Parkinson’s disease. Adv Protein Chem Struct Biol.

[58] Aoki K, Takeuchi T, Itoh I (1994). Clinical significance of anti-cardiolipin antibody in patients with systemic lupus erythematosus (SLE). Ryumachi.

[59] Oostenbrug LE, Drenth JPH, de Jong DJ (2005). Association between Toll-like receptor 4 and inflammatory bowel disease. Inflamm Bowel Dis.

[60] Takeuchi O, Akira S (2010). Pattern recognition receptors and inflammation. Cell.

[61] Xie H, Sheng L, Zhou H, Yan J (2014). The role of TLR4 in pathophysiology of antiphospholipid syndrome-associated thrombosis and pregnancy morbidity. Br J Haematol.

[62] Liu B, Yang Y, Dai J (2006). TLR4 up-regulation at protein or gene level is pathogenic for lupus-like autoimmune disease. J Immunol.

