# Supplementary material for: Saturation of acyl chains converts cardiolipin from an antagonist to an activator of Toll-like receptor-4
Source: Cell Mol Life Sci. 2019 May 6;76(18):3667–78. doi: 10.1007/s00018-019-03113-5 (PMC6697720; doi:10.1007/s00018-019-03113-5)
Supplement: Supplementary file 1 — Supplementary material 1 (PDF 695 kb) [file 18_2019_3113_MOESM1_ESM.pdf]

Supplementary figures

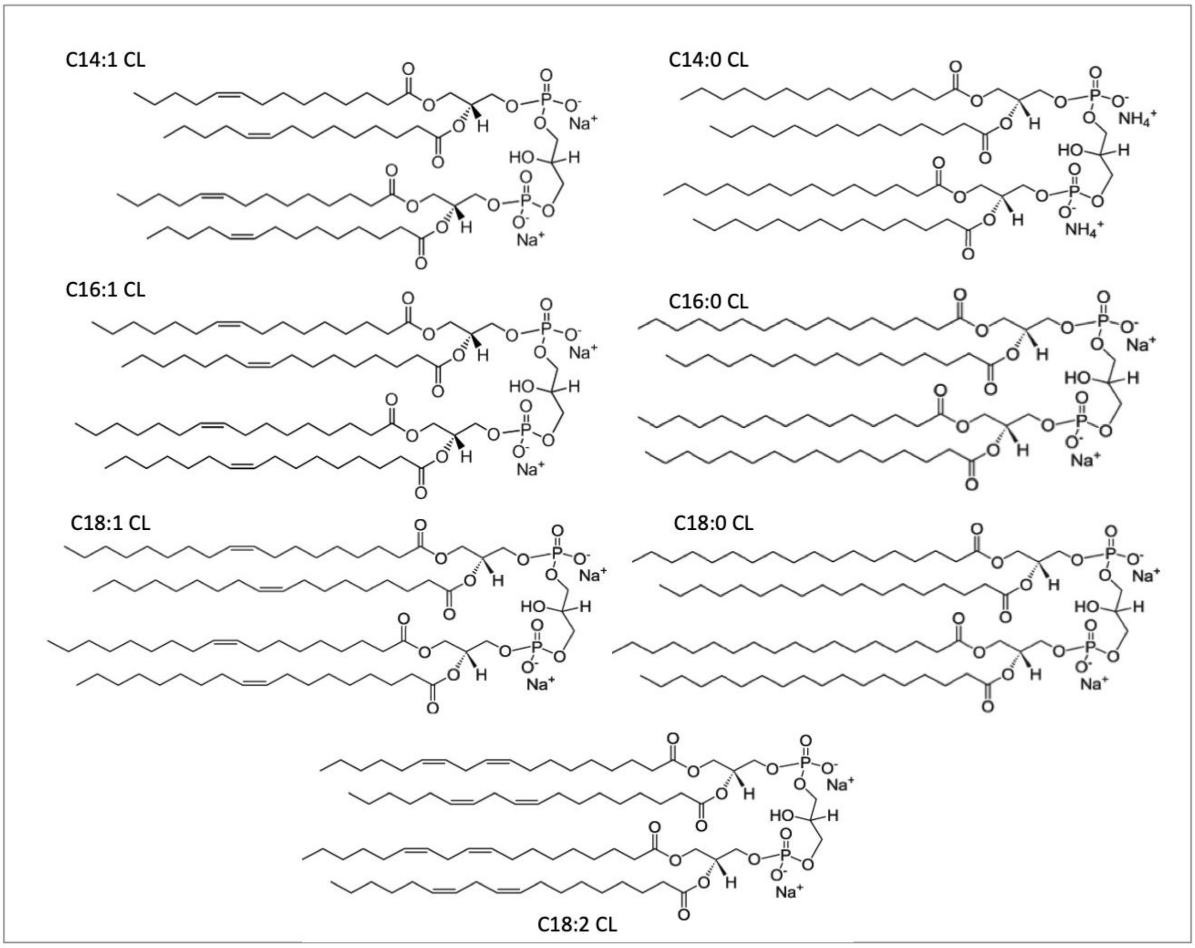

Figure S1: Structure of $(C14:0)_4$, $(C14:1)_4$, $(C16:0)_4$, $(C16:1)_4$, $(C18:0)_4$, $(C18:1)_4$ and $(C18:2)_4$ cardiolipins

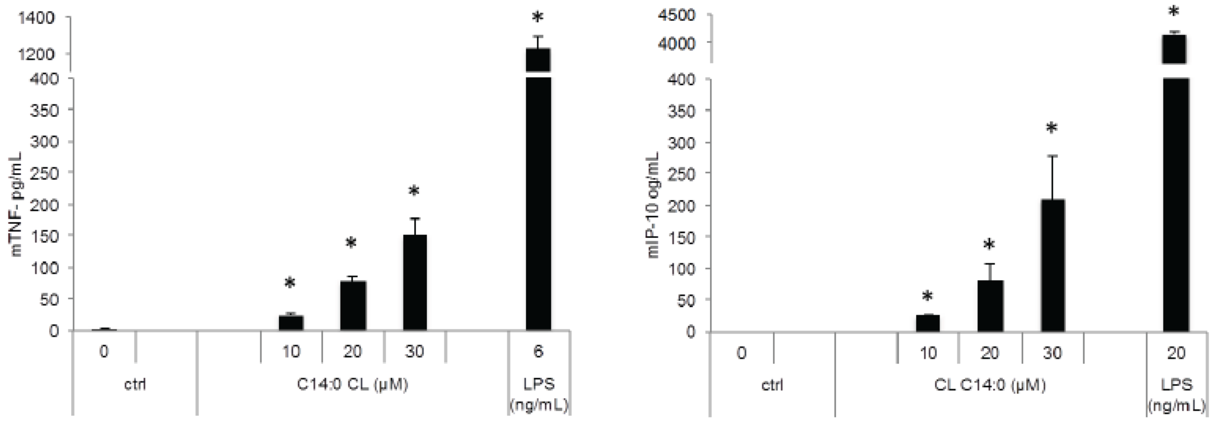

Figure S2: C14:0 CL induces TNF-α and IP-10 secretion in primary BMDM cells.

Primary BMDM cells were incubated overnight with medium (ctrl) or C14:0 CL at the indicated concentrations. TNF-α and IP-10 were measured by ELISA in collected supernatants. Unpaired t-test: differences to respect to the Ctrl are not significant if p>0,05(no symbol); *p<0.0001 Each bar represents the mean + standard deviation of three biological replicates (n = 3). The experiment is representative of 3 independent replicates.

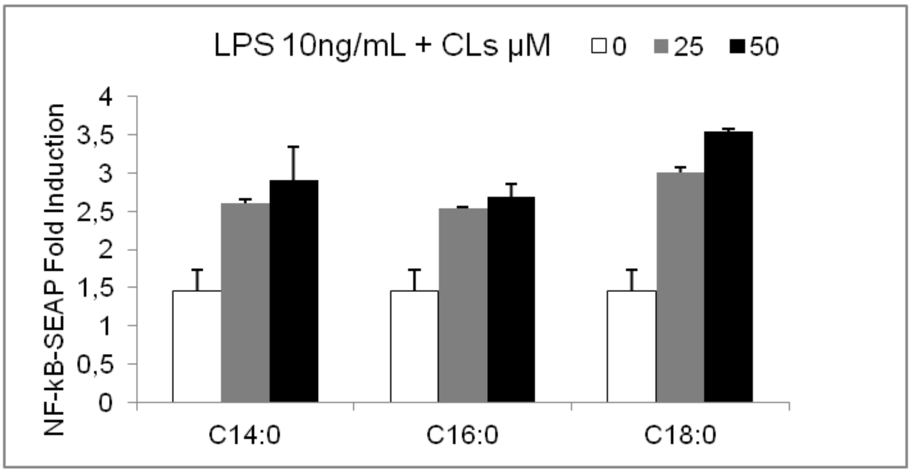

Figure S3: Saturated CLs do not inhibit low LPS concentration

Raw-blue cells were co-incubated overnight with LPS 10ng/mL and the indicated amount of CL (µM). Then NF-κB activation was quantified via Quanti-Blue test (SEAP assay).

Hypothesis of the structural basis of CL/TLR4 interaction

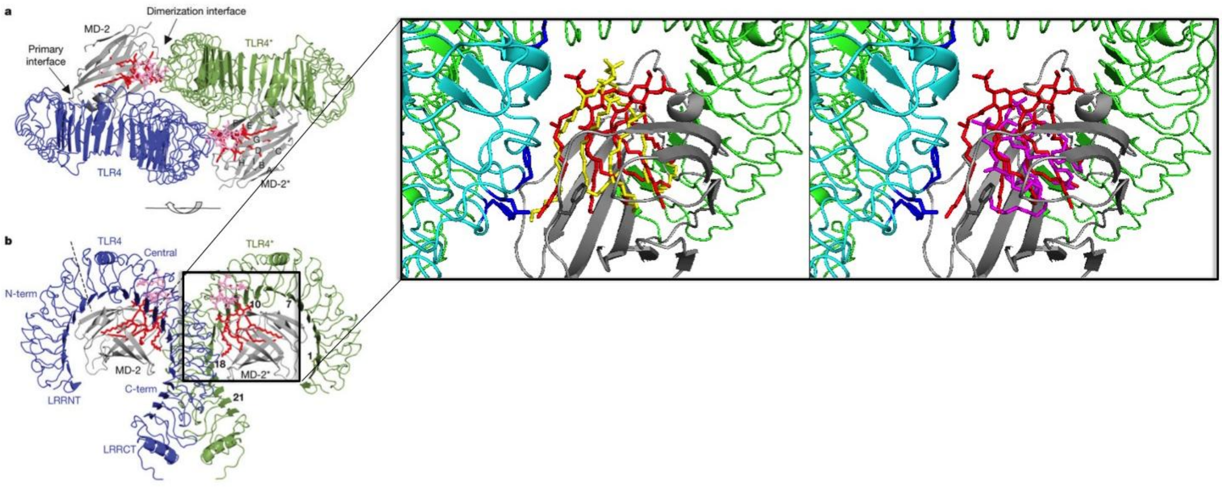

Figure S4: Hypothesis of the structural basis of CL/TLR4 interaction: Overlap of CLs on crystal structure of lipid A/TLR4/MD2

Red= Lipid A(LPS); Yellow= agonist saturated 18:0 CL; Magenta= antagonist unsaturated 18:2 CL; Grey=MD2*; Cyan=TLR4; Green=TLR4*; Blue:TLR4 key contact residues at the dimeric interface.

Because of the cis double bonds, antagonist C18:2 CL is deeply buried in the MD-2* pocket and does not interact with TLR4* nor TLR4 which explains its antagonistic activity. In contrast C18:0 CL is likely positioned as high as lipid A, and contacts key residues at the dimeric interface, which explains its agonistic activity.

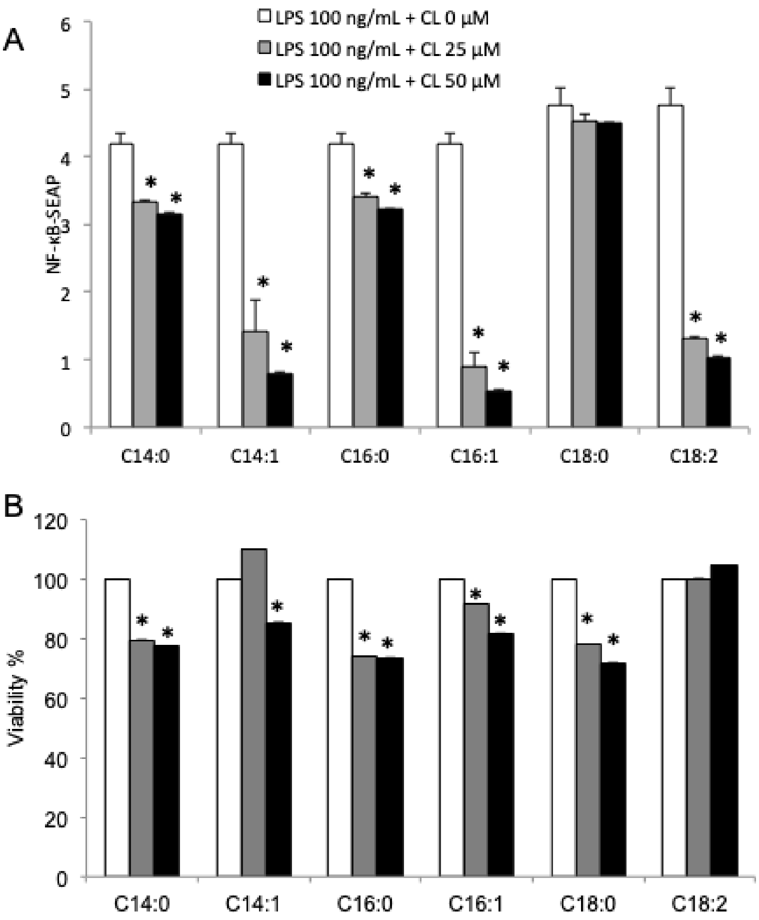

Figure S5: Unadjusted and viability data related to figure 2A.

murine macrophages RAW-Blue™ cells were incubated overnight with LPS 100ng/mL alone (+ CL 0 µM) or together with the indicated amount of each CL (25 or 50 µM).

A) NF-κB activation was quantified in collected supernatants via Quanti-Blue test (SEAP assay) and reported here as fold induction to respect to untreated cells.
B) After supernatants collection, cells were incubated 3 hours with 0,5 mg/mL of MTT diluted in medium without phenol red. MTT, a yellow tetrazole, is reduced to purple formazan crystals in living cells. Plates were centrifuged, medium discarded and crystals diluted in DMSO. Absorbance at 570nm (corresponding to formazan crystal absorbance) and 620nm (used as a reference) was read with a BioTek Synergy HT Microplate Readers and reported here as the percentage of the values measured for untreated cells.
A,B) Graphs are representative of at least three independent experiments. Unpaired t-test: decrease compared to LPS is not significant if p>0,05 (no symbol); *p≤0,002.

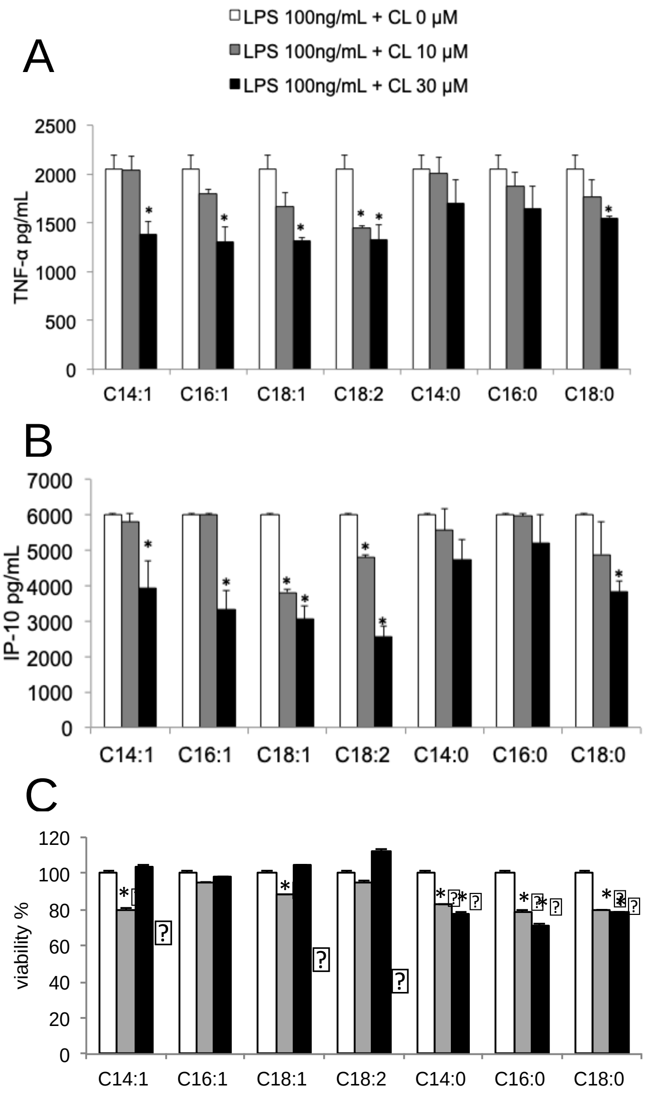

Figure S6: Unadjusted and viability data related to figure 2C and D.

Primary BMDMs were incubated overnight with LPS 100ng/mL alone or together with the indicated amount of CLs.

A and B) TNF-α (A) and IP-10 (B) were quantified in collected supernatants by ELISA assays.

C) After supernatants collection, cells were incubated 3 hours with 0,5 mg/mL of MTT diluted in medium without phenol red. MTT, a yellow tetrazole, is reduced to purple formazan crystals in living cells. Plates were centrifuged, medium discarded and crystals diluted in DMSO. Absorbance at 570nm (corresponding to formazan crystal absorbance) and 620nm (used as a reference) was read with a BioTek Synergy HT Microplate Readers and reported here as the percentage of the values measured for untreated cells.

A,B,C) Unpaired t-test to respect to LPS: decrease is not significant if p>0,05 (no symbol); *p≤0,05. Each bar represents the mean + standard deviation of three biological replicates (n = 3). Graphs are representative of at least two independent experiments.

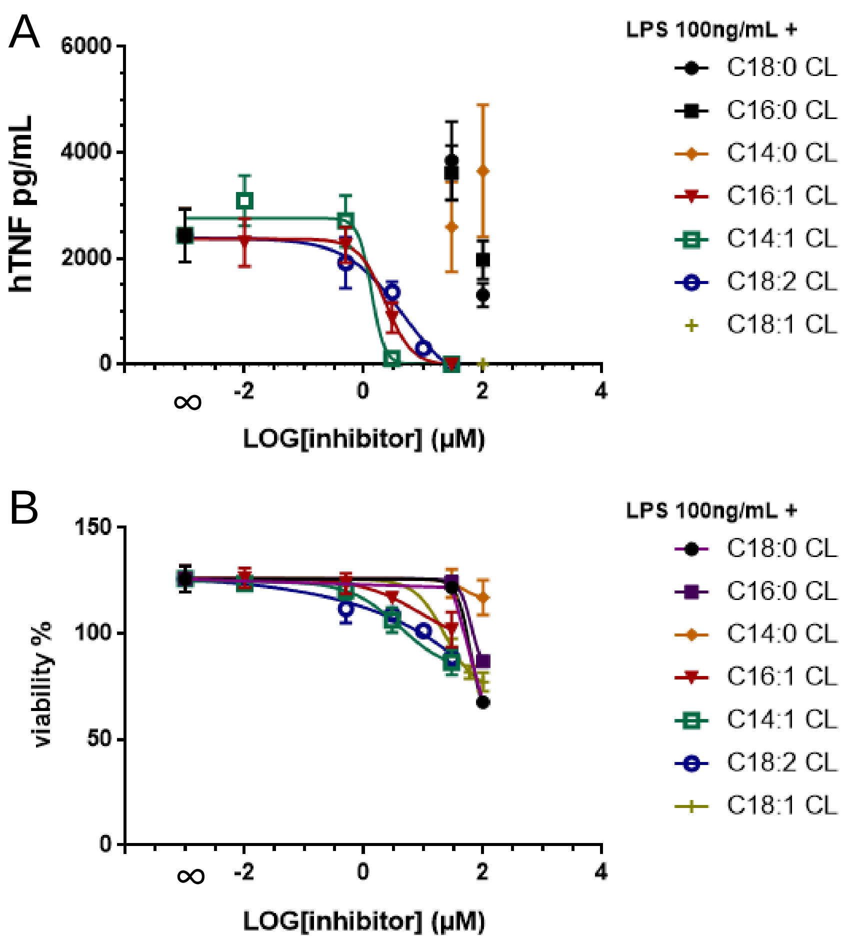

Figure S7: Unadjusted and viability data related to figure 2B.

Primed THP1 cells were incubated 1 hour with the indicated amount of each CL, washed and incubated 5 hours with 100 ng/mL of LPS.

A) TNF-α was quantified in collected supernatants by ELISA assays

B) After supernatants collection, cells were incubated 3 hours with 0,5 mg/mL of MTT diluted in medium without phenol red. MTT, a yellow tetrazole, is reduced to purple formazan crystals in living cells. Plates were centrifuged, medium discarded and crystals diluted in DMSO. Absorbance at 570nm (corresponding to formazan crystal absorbance) and 620nm (used as a reference) was read with a BioTek Synergy HT Microplate Readers and reported here as the percentage of the values measured for untreated cells.
